# Acute and Chronic Tonsillitis

**Published:** 1889-02

**Authors:** E. J. Lee


### ACUTE AND CHRONIC TONSILLITIS.

In the treatment of this affection, it is always our desire to abort those cases which are seen early enough to make this possible; and in cases seen too late for the accomplishment of this desirable end, to at least prevent any profuse suppuration. Of this disease, Flint says: "Acute tonsillitis generally ends in suppuration; an abscess forms, and purulent matter, sometimes fetid, and nauseous to the taste, is discharged after a period varying in different cases from two to ten days."

Many persons seem to be predisposed to this affection, and have their tonsils more or less swollen and inflamed all the time; others suffer from a chronic enlargement of the glands without much pain or discomfort; sometimes this swelling is so great as to threaten the respiration. We have, then, two purposes in our treatment of this disease, one to prevent and to ward off the constant recurrence of these attacks, and to cure the predisposition to this chronic swelling; the other, to cure as quickly and as easily as possible the acute attacks, aborting the inflammation whenever possible.

In prescribing for this, as well as all other disease, we must remember to take into consideration all the symptoms of the patient—that is, we must consider both the symptoms of the disease and of the patient. The disease presents symptoms which are common to nearly all cases of that disease; the patient presents symptoms which are peculiar to the one individual now suffering from that disease. The true homœopathic simillimum should, therefore, cover the symptoms of the disease plus those of the patient. Let me illustrate this proposition by supposing we have a case in which the individual symptoms are covered by a drug which has not the symptoms of the disease, we then search further until we find a drug which covers both features of the case; neither would we give a drug which covered only the symptoms of the disease and failed to cover those of the individual; this would be a very grave error. We should strive to cover all the symptoms of the case, and if a choice had to be made between the generic symptoms of the disease and the peculiar symptoms of the patient, then preference should always be given the peculiar and individual symptoms of the patient.

Tonsillitis is an inflammation of the glands accompanied by more or less inflammation of pharynx; great soreness on swallowing, or talking and opening jaws, or moving the neck; sometimes with fever, headache, flushed face, even convulsions in children. These are the symptoms of tonsillitis as it is generally met; they are the symptoms of the disease, and vary only with the severity of the attack. The symptoms of the patients, on the other hand, are very variable, differing as they do in each person, and these are the most important symptoms in deciding our choice of the proper remedy. In a dozen cases of tonsillitis of equal gravity, the symptoms of the disease would not vary much; but in this dozen cases the individual (and hence the peculiar) symptoms of the patient would vary in each case. In prescribing, it is our duty to find out these peculiar symptoms; they will be found in each case if searched for carefully and diligently. Physicians who do not find these peculiar symptoms, and say they do not exist, are the ones who do not cure tonsillitis. They claim that one must resort to anodynes, to poultices, and finally to the knife.

In considering the homœopathic therapeutics of tonsillitis, we shall confine our attention almost entirely to the local therapeutics; if we considered all the many symptoms which might be concomitant to this or any other disease, we would simply have to take into our paper the whole materia medica. Even when thus confining our work, we must of a necessity speak of many symptoms which belong to diphtheria, angina, etc. Jahr's advice is too brief and of a routine character, but we give it for what it is worth!

He advises the use of Aconite whenever the case is ushered in with fever, dry skin, restlessness, etc.; next, he changes to Belladonna, if the patient complains of a good deal of headache, and rush of blood to the head. If this does not help, give, according to circumstances, *Hepar*, if the pains during deglutition are very severe, glancing and dart to the ear and cervical glands, with severe drawing pains in the nape of the neck; or *Lachesis* if the neck is very sensitive to the least touch, and the symptoms are much worse, after the patient wakes from sleep; or *Silicea*, if the throbbing and lancing pains and the swelling of the tonsils continue to increase in spite of *Bell.* and *Hepar.* If an abscess begins to form, which *Bell.* had been unable to prevent, at once resort to *Merc.*, which generally causes the abscess to discharge in less than twenty-four hours, but which must never be given prematurely; for, if the abscess is not yet sufficiently ripe, this agent often increases the inflammation and renders it more obstinate. If the tonsils become indurated, *Ignatia* often helps, which will likewise be found indicated by flat, open ulcers on the tonsils; although ulcers that break out rapidly and spread extensively, most commonly require *Bell.*; slowly-arising and rather painless ulcers finding their chief remedy in *Merc.* If in this kind of phlegmonous angina with swelling, the velum palati is swollen rather than the tonsils, prefer *Phosph.*, *Arsen.*, or *Bry.*, if neither *Acon.* nor *Bell.* helps; or, if the uvula is the most swollen part, give *Coff.* or *Laches.* Chronic swelling of the tonsils requires particularly *Baryt.*, *Sepia*, *Sulph.*, or *Calc.* Aphthous angina faucium. These inflammations are characterized by small, whitish, flat ulcers on the tonsils; if they are not soon relieved by *Ignat.*, *Merc.*, and *Carbo-veg.*, *Nitr-ac.* is often an indispensable remedy; likewise *Caps.* in many cases, especially if the ulcers burn, with pressure in the fauces as if caused by spasm. We will now take up each remedy in detail, and give its chief symptoms bearing upon tonsillitis:

ACONITE is seldom called for in this disease but may be needed in cases caused by exposure to cold, dry winds, exhibiting the fever, the restlessness, and anxiety of this remedy, together with dark-red swelling of the parts; pricking, burning in the throat and along the eustachian tube, compelling the patient to swallow. Stinging pains when swallowing. Pains worse at night, when they seem to become unbearable.

AMMONIUM-CARBONICUM. Under this remedy we find these symptoms: Pain in the throat as if right tonsil were swollen when swollowing. Burning in throat; tendency to gangrenous ulceration of the tonsils; tonsils enlarged, bluish, and much offensive mucus. Symptoms worse night and morning. These symptoms are frequently met with as concomitants of grave cases of scarlatina.

The symptoms of Amm-carb. seem to be often given as those of Amm-mur.; both Johnson and Lilienthal give under Amm-mur. this symptom: "Both tonsils swollen, patient can neither talk, swallow, nor open mouth, after taking cold." Dr. Johnson adds, "tendency to gangrenous ulceration." Hering tells us that the tonsils and glands of the neck throb but are not swollen. Farrington says: "The throat is swollen so that the patient cannot open his mouth. The mouth is filled with a viscid phlegm, which the patient expels with great difficulty." The true symptom of Amm-mur. seems to be about thus: The throat, inside and out, is swollen and is very sore, with much viscid phlegm in the mouth, which cannot be easily expelled; the tonsils and glands of the neck throb, there is also uneasiness and anxiety.

AMYGDALA PERSICA. Of this remedy Farrington said: "This drug causes a dark-red injection of the fauces, uvula, and tonsils, with sudden, sharp pains, causing considerable difficulty in swallowing; sometimes these pains are so severe as to make the patient cry out."

APIS. The pains of this remedy are burning, stinging, often accompanied by dryness without thirst. The tonsils are inflamed, swollen, and bright red, smarting or stinging on swallowing. Sometimes we see deep ulcers on the tonsils and palate, with sloughing abrasions, and an œdematous or erysipelatous appearance around them. Swallowing is difficult, especially of solid, hot, or sour substances. Worse from heat or hot drinks, better from cold and cool drinks. The restlessness, the raw, scalded feeling in mouth and throat with the puffy swelling, are pecular to Apis.

AURUM, has red, swollen, and ulcerated tonsils; after abuse of Mercury or from syphilis.

BAPTISIA. The indications for this drug are these: Putrid ulceration, with salivation; tonsils and parotids swollen, with but little pain; fauces dark red. Tonsils and soft palate very red and swollen, with constant desire to swallow; no pain. Patient can swallow only liquids, the least solid food gags. (This dysphagia of solids is also found under Baryta, Bell., Bry., Ign., Lach., Lyc., Natr-m., Stram.) The patient will generally be found in a typhoid state with this condition of mouth and fauces, and we are apt to wonder that such a bad-looking throat causes so little pain.

BARYTA-CARB. In every case of this disease, where a predisposition to the disease seems to exist, we think of this remedy, and it will not disappoint us if used properly. In acute tonsillitis or in chronic indurations of the tonsils, occurring after every little exposure to cold, or after checked foot-sweat, with these symptoms, we expect to relieve with Baryta.

The tonsils are inflamed and swollen, with smarting pain when swallowing ; is worse when swallowing food or saliva. Sometimes cannot swallow at all and fluids will be ejected through the nose. Symptoms going from right to left. (It is well to remember that under Lachesis we find this ejection of fluids through the nostrils, also under Lyc., but the Lachesis patient swallows solids easier than either fluids or simple saliva ; Lachesis and Sabadilla have their throat symptoms going from right to left.) Under Baryta, we also find a sensation as if the food on being swallowed passed over a sore place ; also a feeling as if there were a morsel of food lodged in the throat. Baryta is the better indicated if the patient be of a scrofulous habit ; or if a child, it is dwarfish, or in old fat people. The patient has viscid phlegm in mouth in mornings, is thirsty. Hering tells us that the Baryta throat is much paler than that of the Belladonna patient, and that in cases where the tonsils are inflamed in small-pox or scarlatina, especially when Mercurius has failed, then Baryta may be needed.

BARYTA-MUR. is also often indicated in scrofulous persons with chronic enlargement and induration of the tonsils ; there is profuse salivation, the pain seems to be worse on the right side.

BELLADONNA. Dryness of mouth and pharynx, with sense of constriction and difficult swallowing, especially of liquids or saliva. Constant burning and pressing. Deep crimson color of the throat and enlarged tonsils, with throbbing ; worse on the right side (also Baryta) and on swallowing. Soreness extending to ears. Rapidly forming aphthous ulcers on tonsils, intense congestion and throbbing of carotids. During deglutition there is a sensation as if the throat were too narrow, and as if nothing would pass properly. Externally the throat is painful to touch and on motion. Constant inclination to swallow or to hawk. The peculiarities of Bell. are its sudden pains, deep redness and throbbing, and its signs of intense active congestion.

BROMINE may be called for in the chronic forms of tonsillitis in persons of a scrofulous habit, blue eyes, and fair complexion. There is constant pain in the throat, the tonsils are deep red and swollen, with a net-work of dilated blood-vessels spread over them (see Ham.); the right side of fauces dark red and dry with pain on swallowing. Scraping in throat. Aggravation on swallowing, especially of liquids. This condition will generally be accompanied by swelling, hard swelling, of the external glands.

CALCAREA in persons of a psoric habit, who exhibit the peculiar general symptoms of this remedy with the inflammatory swelling of palate and uvula or tonsils, with a sensation as if the throat were constricted on swallowing. Ulceration of tonsils. Pains in throat extend to the ears. Sensation of swelling or of a lump in the throat cause constant desire to swallow (see Plumb.).

Calcarea-iod. and Calc-phos. may also be needed in the chronic form of this complaint; in the latter the throat does not pain so much on swallowing food or warm drinks as from saliva.

CANTHARIS, intense burning, burning as from fire (see Iris.); sometimes with a scraped sensation and spitting of blood. Aphthous ulcer at back part of fauces, covered with a whitish adherent crust; also on right tonsil. Tonsils inflamed or suppurating; swallowing very difficult. Throat symptoms are worse at night, when drinking, and from wet poultices; are better when lying down. Especially indicated when accompanied by the urinary symptoms of this drug.

COLCHICUM. Throat is dry yet there is a flow of watery saliva, with nausea and discomfort in abdomen. Tonsils inflamed and swollen; here and there spots covered with pus; swallowing is difficult.

COCCULUS has a pressive pain in tonsils, worse when swallowing saliva than when swallowing food. Burning in palate and dryness in fauces. Choking constriction in upper part of fauces, with difficult breathing and irritable cough or disposition to cough.

CROTALUS. Quinsy, with much venous congestion, dark, bluish color of surrounding parts, much œdema, tonsils bulge and are tender to pressure at angles of lower jaw; pain worse from empty deglutition. Especially indicated when occurring with scarlatina or diphtheria. Also angina tonsillaris, constriction of throat, tongue yellow. Great prostration, etc.

CUPRUM, sense of constriction in throat. Tonsils, palate, and fauces red and inflamed; dull, piercing pain in left tonsil, aggravated by touch.

CYCLAMEN presents an opposite condition to enlargement, etc., of tonsils, but may, nevertheless, be mentioned here. Under this drug the tonsils and palate are shriveled and white.

DIOSCOREA. In the last number of THE HOMŒOPATHIC PHYSICIAN, Dr. J. B. Bell calls our attention to the resemblance of this drug to the first stage of many colds, premising that it may be found of use in some of these cases. We find, under this drug, *dryness, soreness, smarting, and burning* in the whole throat. Left tonsil smarts and itches, with inclination to cough. Sharp, stitching pains, seemingly in the tonsil to ear. Mouth dry, yet full of sticky mucus; no thirst.

DULCAMARA probably covers some of the cases for which Baryta has been given, and failed. For it, too, has a tendency to "tonsillitis" from taking cold. But, under Dulcamara, we find the patient is affected by every cold change in the weather (see Hepar), as well as by actually taking cold. We have, under this drug (like Gels.) catarrhal angina, hyperæmia of soft palate and uvula, swelling of tonsils, difficult deglutition. Constant hawking of very tough saliva, with rawness in the fauces. Baryta and Dulcamara are complementary to one another.

ELAPS. Deglutition of solids and liquids almost impossible; throat exceedingly sensitive to touch (like Lachesis and Lac-can.); tonsils swollen so that no passage is visible. Pain goes to ears when swallowing. Nasal discharge, when present, is very offensive, smelling like putrid herring brine. Aggravation from wet weather; never feels happy in wet weather. Elaps has a peculiar throat symptom, which may sometimes be noticed as a concomitant of its throat or nasal diseases; it is this: The posterior wall of the pharynx is covered with a dry, greenish-yellow membrane, wrinkled and fissured, extending into nares; sometimes portions of this membrane are expelled from nose or mouth, leaving a raw, corrugated surface.

FERRUM-PHOSPHORICUM. Under this remedy, we find constant inflammation of palate, tonsils, and pharynx, with dryness, redness, and pain. Pulse full, round; fever, red face, glistening eyes, etc.; inflammation from relaxation of the blood-vessels, before any pus is formed.

FLUORIC ACID. In cases where syphilis is a probable factor, with these symptoms, Fluoric acid may be useful We find tonsils, uvula, and soft palate of a livid color, and greatly swollen. Excessive suffering on swallowing or talking. Sleep is disturbed by the accumulation of mucus in the fauces. The throat is irritable, and particularly sensitive to cold; the slightest exposure causes inflammation, with pain and impeded deglutition. Tongue tender, and pains when talking. A peculiarity of this remedy is the lack of susceptibility of the patient to the extremes of heat and cold; yet we find aggravation from cold, wet weather.

Gelsemium in cases of catarrhal inflammation of pharynx and tonsils; dryness, and burning in the throat; throat feels filled up; chilly creepings up the spine; headache, fever, aching in back and limbs, etc. Sometimes the tonsils are swollen, and covered with diphtheritic patches.

Graphites has swelling of tonsils, with pain when swallowing. Also roughness and rawness in throat. The peculiar symptom of this drug, in this connection, is the feeling as though food could not be swallowed; it "will not go down." On swallowing, there is always a sensation as of a lump, or plug, or elevation in the throat, which prevents the passage of food or saliva. (With Hepar, there is pressure or swelling, which causes fear of choking.)

Guarea-tri. Swelling of tonsils, rendering swallowing difficult. Sensation of constriction and burning in throat. Warm drinks ameliorate throat symptoms. This remedy is to be compared with Merc. and Silicea in bone pains and suppurations.

Gymnocladus. Inflammation, and purple color of right tonsil. Sore throat, dark livid redness of fauces and tonsils. Mucus in throat, and frequent hawking. Shooting, sticking pains in throat. Aversion to motion.

Hamamelis. Hering tells us this remedy is useful for the sore throats of those predisposed to fullness of their veins, with aggravation in warm, moist air. We find sore throat worse on right side; right tonsil more swollen than left, reddened, and veins enlarged. (See Bromine and Crotalus.) Tonsils and fauces congested; the parts look bluish from the distended veins.

Hepar-sulph., chronic tonsillitis, especially when accompanied by deafness (see Kali-b.), or by a sensation of sticking in throat, as if from a fish-bone, or splinter (see Nitr-ac.) when swallowing; has a tendency to suppuration, after a week or two; tonsils swollen so as to leave no opening visible; swollen with hard glandular swellings on the neck (like Bromine). Hering gives this case: Tonsils enlarged, red; throat and pharynx raw, and studded over with enlarged reddish follicles; could not venture out in the slightest damp weather (like Dulc.) without being in fear of inflammation of the throat, which at last produced a nervous terror of being choked. This patient could not work in damp clay, as the dampness affected him with hoarseness and irritability of the chest. Especially useful for patients who have been abused by large doses of Mercury. (Also Staph.)

IGNATIA. Follicular tonsillitis. Acute paroxysms in chronic cases, with a feeling of swelling in the throat; painful soreness during deglutition. Inflamed, hard, swollen tonsils, with small ulcers. Whitish, tough mucus in spots on tonsils, simulating diphtheria. Tonsils greatly swollen and inflamed; several small openings filled with pus; stitching pains in throat. The sticking pain of this drug is peculiar; it occurs when not swallowing (see Ledum), also somewhat when swallowing; but the more he swallows, the more it disappears; swallowing anything solid, like bread, seems to cause the sticking to disappear entirely. Patient is chilly, is despondent, tearful, etc.

INDIUM-MET. may be called for in cases where there is destructive ulceration of tonsils, uvula, and soft palate, with thick yellow mucus in the ulcers. There is dryness, throbbing, stinging soreness in the throat; apt to be worse on the right side; worse on swallowing. Uvula greatly enlarged; back part of pharynx covered with thick yellow mucus, very tough, and hard to remove (see Elaps). Throat symptoms are worse in the evening; are better after eating, and from drinking cold water.

IODIUM must not be forgotten in cases where the tonsils are swollen, and are covered with little patches of exudation. The palate and tonsils are covered by a thick, grayish-white exudation; there is much pain in the throat, very painful deglutition, some salivation, very offensive odor from the mouth; the external glands of the neck are swollen. These are rather the symptoms of diphtheria than of tonsillitis, but as the two often resemble one another, they may be appropriately mentioned in this connection.

IRIS-VERS. In a patient whose mouth and fauces felt as if on fire (see Canth.), or as if they had been scalded; from whose mouth there was a constant discharge of saliva, maybe a ropy saliva; who complained of a smarting, burning in throat, with a feeling of enlargement, like a burning cavern (see Phyt.), while his throat was dry, injected, and of a bright-red color, also pain in tonsils shooting to the ears.

JACEA for cases of syphilitic ulcers, where there is a prominent yellow-greenish ulcer, with adherent pus, in left side of throat, extending from velum palati over the entire left tonsil. Much phlegm; swallowing is very painful.

KALI-BICHROMICUM is especially useful in syphilitic or diphtheritic affections of the throat. We find recorded under this drug these symptoms: Swollen tonsils, with deafness in children (see Hepar) Tonsils swollen, neck thick below the angle of the lower jaw (see Crotalus); the eustachian tubes seem to be blocked up; is very deaf, could not hear a watch ticking unless very close to the ear. Also sharp, shooting pains in left tonsil, extending toward ear, better after swallowing; suppuration of tonsils. Indolent enlargement of tonsils, where there is little fever or inflammation, but there is a tendency to the formation of small ulcers on tonsils and the velum (something like Ign.). The ulcers on the tonsils and throat seem to be covered with an ashy slough; the surrounding mucous membrane is dark, livid, and swollen. The uvula and tonsils are red, swollen, and painful; finally become ulcerated. There is hawking of much tenacious mucus, which is difficult to get up; is so very stringy that it sticks. The throat pains are worse when protruding the tongue (see Sabad.), and are generally aggravated on swallowing. The ulcers of this remedy eat deeply and quickly.

KALI-BROMATUM. With this drug the tonsils are swollen and purple; the exudation (diphtheritic) is thick, and looks something like patches of washed leather; there is a distinct, but crooked line of demarcation between the healthy and the affected tissue. There is often dysphagia of liquids; patient can only swallow solids (see Lachesis).

KALI-MURIATICUM. Catarrh of the mucous membrane of the fauces, tonsils, and pharynx, with a white exudation. Angina beginning with white points on the openings of the ducts of the glands; fever, chilliness, dirty, coated tongue, suffering expression of the face. Tonsillitis, with much swelling. Tonsils swollen, and covered whitish or whitish-gray. Hawks up little cheesy lumps having a disgusting odor and taste.

LAC-CANINUM. Tonsils inflamed and very sore, red and *shining;* almost closing up the throat; dryness of the fauces and throat; swelling of submaxillary glands. Also right tonsil red and swollen; pain in the tonsil of a gnawing kind, worse at night, and after sleep. The pains and the inflammation of this remedy continually change from side to side, and back again. The sore throats begin with a tickling sensation, which causes a constant cough; then comes a sensation of a lump on one side, causing constant deglutition; this condition then ceases entirely, only to begin on the opposite side, and often alternates, returning again to its first condition; with women, these sore-throat symptoms are very apt to begin and end with the menses. Suppuration begins in one tonsil, and then in the other, finally returning in the first one, etc. The exudation is generally of an ashy-gray color. Aggravation on swallowing. Externally, the throat is sensitive to touch, like Lachesis and Elaps; there is aggravation from empty swallowing, like Ignatia.

LACHESIS. Swollen, congested tonsils, with a yellow, small patch on each; great difficulty in swallowing, with constant desire to do so; pain begins on the left side, goes to the right, and upwards toward ear on swallowing; heat and chills alternating. Sensation of fullness and rawness in throat; frequent desire to swallow, which causes pain, extending deep into the ear; fluids are ejected through the nose, with great fear of suffocation; gums, tonsils, and uvula dark-red and swollen, the latter looks as if squeezed and crowded back; large collection of mucus in the mouth, which forms large bubbles when the mouth is opened. Aggravation after sleep, from hot drinks, and from the slightest touch, cannot bear even the sheet to touch his neck. Chronic enlargement of tonsils. Hering says there is no remedy so effective either for aborting tonsillitis, or for promoting resolution in later stages.

(Dysphagia of liquids is found under Bell., Brom., Bry., Canth., Hydro-ac., Hyos., Ign., Kali-brom., Lach., Lyc., Podo. Dysphagia of saliva, Calc-ph., Cocc., Ign., Lach., Merc., and Crotalus.)

LYCOPODIUM. Under this drug we find almost an opposite condition from that presented by Lachesis; the symptoms go from right to left, and are generally aggravated by cold drinks, especially by other cold drinks than water.

It is useful, when properly indicated, in cases of ulcerated tonsil, or for chronic enlargement of the tonsils. We find soreness of the throat commencing on the right side, going to the left side, with whitish ulcer on right tonsil, also tonsils studded with many small ulcers, sharp pain on swallowing, especially cold drinks; pain, as if bruised, all over limbs; frontal headache; sometimes a sensation when swallowing as if the head opened, and as if a pain shot down into the abdomen. Stitching pain, with sensation as if a hard body had lodged in back part of throat. Inflammation and enlargement of tonsil, with yellow, small patches on each tonsil. Although this drug generally has an aggravation from cold drinks, it has also a smarting pain in throat from hot drinks; this should be remembered as an exception to the rule. If the case be a severe one, we will find great prostration, fan-like motion of the alæ nasi; dyspnœa, etc.

MANCINELLA has great swelling and suppuration of the tonsils, with danger of suffocation; whistling breathing. White, yellowish ulcers on tonsils and in throat; with burning pain. Thirst for cold water, but unable to swallow on account of a choking which rises up from the stomach; this choking sensation also rises when speaking. The breath, when offensive, is noticed by the patient.

MERCURIUS, soft palate, and tonsils greatly swollen, dark, coppery-red, and pressed forward; stinging pains on empty swallowing, at night, and in cold air; worse in fall, spring, and in wet weather. Tonsils enlarged, dark-red, studded with ulcers; mostly useful after pus has been formed to hasten maturation; small, flat ulcers.

MERCURIUS-CYANATUS. The tonsils are greatly inflamed, are dusky-red, and swollen, with whitish spots on them; later, deep ulcers, with yellowish-greenish pus. Right side apt to be the worst. Eyes heavy, fever, headache, and nausea; great redness of the fauces, and difficulty in swallowing; the submaxillary glands are swollen.

MERCURIUS-IOD-FLAVUS. Under this preparation of Mercury we find the tonsils, uvula, and pharynx red and congested; generally worse on the right side; also worse from warm drinks, and on empty swallowing. This case, given by Hering, well illustrates the indications for this remedy: "Stiffness of the jaws, difficult to open the mouth; altered voice, speaks as if had pebbles in the mouth; right side of the throat and tonsil inflamed; soreness in right ear, and over right side of head and face; enlargement of cervical glands; sensation of a lump in right side of mouth; soreness in right ear, extending into throat; pain when swallowing, burning; desire for sour things; hawking; tongue coated yellow at back part, clean in front; later, soreness and swelling attacked left ear and tonsil." Very fretful and restless, as if from pain; refuses to eat or to drink. Cannot sleep. Sometimes the nostrils are dilated with every inspiration.

MERCURIUS-IOD-RUBER. With this preparation the throat symptoms generally begin, or are worse on the left side; are aggravated by swallowing both food and drinks. The patient hawks much; spitting up a tough, white phlegm. Painful swelling of tonsils, and submaxillary glands. The deglutition is painful, with many ulcers in throat; the tonsils suppurate. Patient must breathe with the mouth open. Pain in throat, tonsils swollen, and covered with a slimy, speckled coating; back part of throat red; slight pain on swallowing; prostration; two days later, tonsils, uvula, and back part of pharynx are covered with a coating looking like dried starch.

MURIATIC-ACID. In cases of diphtheria or after scarlet fever, accompanied by great prostration, we sometimes find the tonsils and fauces covered with a dark exudation, the submaxillary glands swollen as large as pigeon's eggs; patient can only hold the head bent forward; continual desire to hawk, with difficult expectoration of tough mucus; swallowing is almost impossible. Œdema of uvula and swelling of tonsils.

NAJA. Right tonsil swollen, with sharp pains in it as from needles, short, hard cough; worse at night; pain up right side of neck; the larnyx is tender to touch, with inclination to cough from any pressure on it. Patient grasps at throat with a sensation as if choking.

NATRUM-ARSENICUM. Tonsils, fauces, and pharynx œdematous and purplish; surface irregular, covered with a yellowish-gray mucus which is hawked out. This chronic case is given by Hering. After an acute attack of tonsillitis, which occurred three months previously, the throat remained very much swollen; the whole fauces and upper part of pharynx swollen and of a dark hue, the tonsils greatly enlarged, the uvula elongated; parts covered with a dirty-looking mucus; constant dry sensation as if something lodged in the throat; at times a feeling as if a pin were sticking in the throat, at others a feeling as of a lump; always worse in the morning. There is prostration, swelling, etc., but not much pain; therein resembling Baptisia.

NITRIC-ACID may be useful in mercurial or syphilitic persons, with red, swollen, uneven tonsils, having small ulcers on them. The ulcers bleed readily, have stinging pains in them, their edges are hard, irregular, and everted. We have also soreness of the palate, tongue (the mildest kind of food causes smarting), and the inside of the gums, with stinging pain and ulceration of the corners of the mouth. Pricking in the throat as from a splinter (like Hepar), worse when swallowing.

PHOSPHORUS. Dryness of the throat, day and night; it fairly glistens. (Under Lac-can. we find the throat shines or glistens very markedly.) Tonsils and uvula much swollen, the uvula is elongated, with dry, burning sensation; mucus in throat, removed with great difficulty, is quite cold as it comes into the mouth. The mucus is white, nearly transparent, and in lumps.

PHYTOLACCA. Tonsils large, bluish, ulcerated; dry, rough, burning, smarting fauces; throat feels like as after a choke-pear. Pharynx dry, rough; feels like a cavern (see Iris). Sensation as of a plug in throat; worse left side. The sore throat is generally worse on the right side; the fauces are dark bluish-red; pain worse on swallowing saliva; sensation as if a red-hot ball had lodged in fauces when swallowing; cannot bear the touch of clothing about the neck; cannot drink hot fluids; is prostrated.

PLUMBUM has inflamed tonsils covered with small, painful abscesses; constriction in throat when trying to swallow with great desire to do so. Angina granulosa going from left to right. Fluids can be swallowed, but solids come back into the mouth.

PSORINUM has tonsillitis with swollen submaxillary glands, fetid otorrhœa; the throat burns, feels as if scalded, pains when swallowing saliva; ulcers on the right side with deep-seated pain and burning in the fauces; mouth is inflamed and sore; worse from warm food, but not annoyed by cold. This remedy is especially useful for pale, sickly children, and for those who have a dirty, greasy, or scaly skin. Body always smells badly.

RANUNCULUS-SCEL. has swelling of the tonsils with shooting stitches in them; there is burning and scraping in the throat. (This sticking pain we have seen occurs prominently under Amygdala, Hepar, Kali-bi., and Nitric-acid.)

RHUS-TOX. Sticking or stinging pain in tonsils, worse when beginning to swallow; the right tonsil is covered with a yellow membrane. Throat sore, feels stiff after straining the throat. Feeling of swelling with bruised pain; erysipelatous inflammation, parotids swollen, cellulitis of the neck, drowsiness.

SABADILLA. Under this drug we find the tonsils swollen and inflamed, nearly suppurating; goes from left to right; stitches in throat only when swallowing. Tonsillitis after coryza; right tonsil remains somewhat swollen and indurated. Cannot swallow saliva on account of the pain, must spit it out. Continual desire to swallow, deeply cutting pains, whole body writhes. There is much tough plegm in the throat, must hawk; also a sensation as of a skin hanging loosely in the throat, must swallow over it; cannot protrude the tongue with sore throat (see Kali-bichr.); can swallow warm food easily; there is often a desire for hot drinks.

SANGUINARIA, we have ulcerated sore throat, burning, especially after eating sweet things; throat feels swollen as if to suffocation, with pain when swallowing and aphonia. Tonsillitis; promotes suppuration.

SILICEA. The tonsils are swollen and each effort to swallow distorts the face. In cases where the suppurating gland does not heal, pus continues to flow, but gets thinner, less laudable, darker, and more fetid.

SULPHUR. Sore throat, great burning and dryness, first right then left side. Swelling of palate and tonsils, elongation of palate. Sometimes the whole back part of pharynx appeared to be in a state of ulceration or sloughing; much nauseous saliva; sometimes needed to aid recovery when after suppuration the parts heal very slowly.

ZINCUM has herpetic-like eruption on tonsils, soft palate, and root of tongue; whitish, somewhat elevated, ulcerated spots in mouth (sequelæ to gonorrhœa), also dryness of throat evening; mucus collects from posterior nares; soreness in throat, tearing in posterior fauces; more between the acts of empty deglutition or after eating. E. J. L.

